# Post-operative pericardial effusion following treatment of small hepatocellular carcinoma with radiofrequency ablation: A case report

**DOI:** 10.3892/ol.2013.1733

**Published:** 2013-12-06

**Authors:** ZHEBO ZHANG, ZHUONAN ZHUANG, ZHENJIE XU, QIANG MEI, KUANSHENG MA, XIAOWU LI, PING BIE

**Affiliations:** 1 Institute of Hepatobiliary Surgery, Southwest Hospital, Third Military Medical University, Chongqing 400038, P.R. China; 2 Department of Hepatobiliary Surgery, Qilu Hospital, Shandong University, Jinan, Shandong 250012, P.R. China; 3 Department of Clinical Laboratory, Rizhao People’s Hospital, Rizhao, Shandong 276800, P.R. China; 4 The Fourth Student Brigade, The Fifth Department of Histology and Embryology, No. 169 Hospital of Chinese PLA, Hengyang 421002, P.R. China

**Keywords:** liver cancer, radiofrequency ablation, pericardial effusion

## Abstract

Radiofrequency ablation (RFA) is a minimally invasive technique used to treat liver tumors. The current study presents the case of a patient with hepatocellular carcinoma who suffered from post-operative pericardial effusion following RFA treatment. We hypothesize that RFA thermal conduction may damage the diaphragm and pericardium, leading to local edema in the pericardium. RFA is a minimally invasive technique, however, adequate preparatory work must be performed prior to surgery, including a comprehensive assessment of the patient. During surgery, the location and extent of the region to receive RFA must be determined precisely in order to reduce the range of damage and to avoid post-operative complications. This study describes a case of pericardial effusion caused by RFA of liver cancer. We analyzed the causes and preventive measures for pericardial effusion in order to contribute to the prevention pericardial effusion that is complicated by RFA of liver cancer.

## Introduction

Radiofrequency ablation (RFA) is an interventional therapeutic method used to treat liver tumors. The technique is effective, minimally invasive, easy to perform and highly suitable for the treatment of patients with primary liver tumors or metastatic liver cancer who are not good candidates for, or cannot receive, surgical treatment (1). For these reasons, RFA has been widely used in clinical practice (2,3). The working principle of RFA is based on a radiofrequency current that causes the conductive ions and polar molecules in the tumor tissue to undergo rapid changes in the direction of the radiofrequency alternating current. The produced frictional heat causes irreversible coagulative necrosis to the tumor tissue itself (2). RFA treatment must be guided by imaging techniques to accurately locate the tumor focus. Common imaging methods for guiding RFA include CT, MRI and color Doppler ultrasound. Currently, color Doppler ultrasound is most commonly utilized as it not only allows the tumor area to be located precisely, but it is also easy to perform, affordable, practical and facilitates convenient, real-time dynamic observations. These traits make color Doppler ultrasound a good method for locating tumors and guiding RFA treatment (4). Common complications observed following RFA treatment for liver cancer include fever, pain, abdominal bleeding, bile duct injury, bowel injury, liver abscesses and implantation metastasis of tumor cells (5). However, to date, there have been no reported cases of post-operative pericardial effusion. The present case report discusses the rare complication of pericardial effusion, which occurred following RFA in a single case. Written informed consent was obtained from the patient.

## Case report

### Patient characteristics

**Figure 1:**
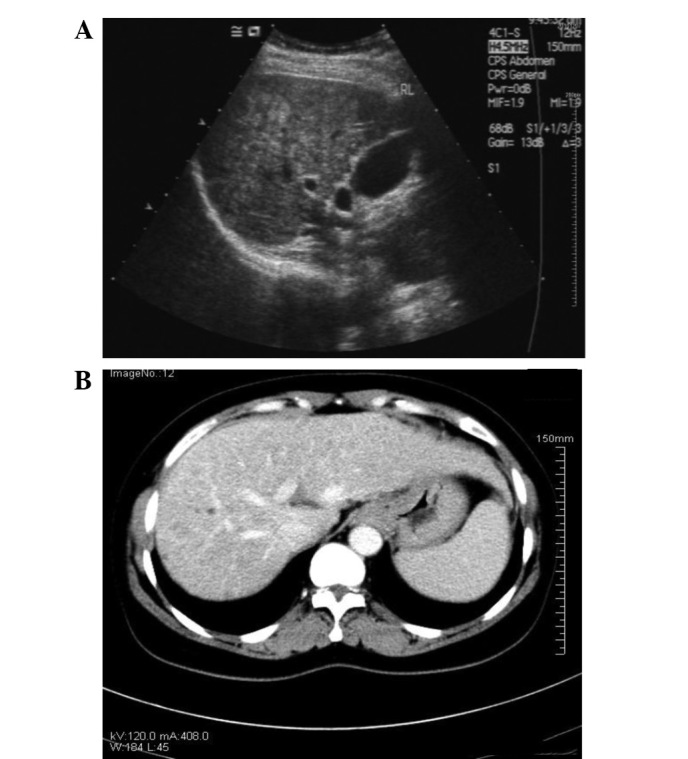
(A) B-mode ultrasound indicating a liver echo change as a solid lump in the left liver; CEUS was consistent with the diagnosis of small hepatocellular carcinoma (HCC). (B) Abdominal enhanced CT indicating multiple space-occupying lesions in the liver. CEUS, contrast-enhanced ultrasound.

A 44-year-old female patient, with a five-year history of hepatitis B, was admitted to Southwest Hospital (Chongqing, China) following identification of a tumor in the left lobe of the liver by ultrasound examination. Following admission, B-mode ultrasound (Fig. 1A) and enhanced abdominal CT (Fig. 1B) were performed. The results were consistent with a diagnosis of liver cancer. The patient’s AFP tumor marker levels of 75.77 ng/ml and the 5-year history of hepatitis B were taken into account and the diagnosis was confirmed as hepatocellular carcinoma (HCC). The tumor was 1.7×1.1 cm in size and located in the left upper hepatic segment, close to the heart.

### RFA treatment

**Figure 2:**
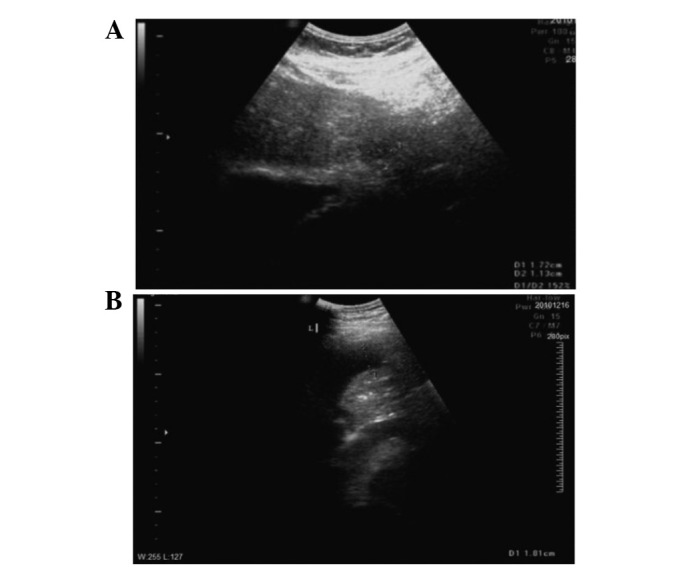
(A) Intraoperative B-mode ultrasound showing a weakened echo in a 17×11-mm area in the left lobe of the liver. (B) Post-operative B-mode ultrasound showing a filling defect in the radiofrequency ablation (RFA) area.

Following discussion, it was decided that the patient met the criteria for RFA treatment. Color Doppler ultrasound-guided RFA was therefore performed. During surgery, B-mode ultrasound showed (Fig. 2A) a 17×1-mm lump in the second segment of the liver. The surgery was performed with no complications. Post-operative contrast-enhanced ultrasound (CEUS) performed following RFA showed that all areas of liver cancer had undergone necrosis (Fig. 2B).

### Post-operative complications

**Figure 3:**
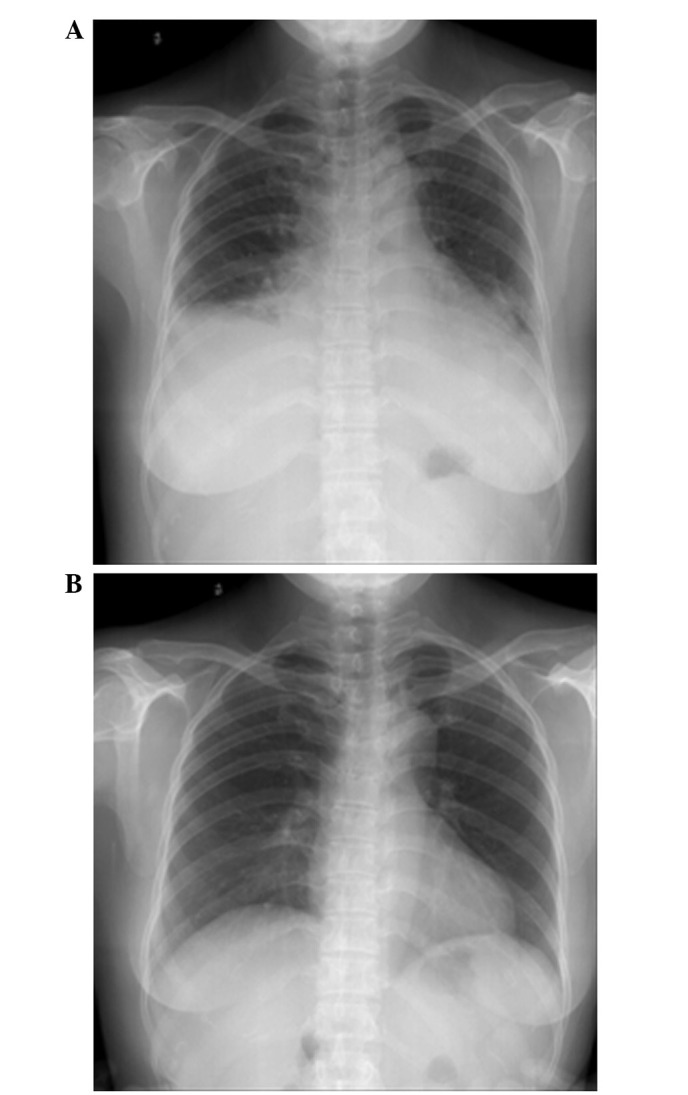
(A) Post-operative chest radiograph showing a small amount of bilateral pleural effusion, infection in the bilateral lower lungs and ardiac enlargement. (B) Pre-operative chest radiograph showing no cardiopulmonary abnormalities.

**Figure 4:**
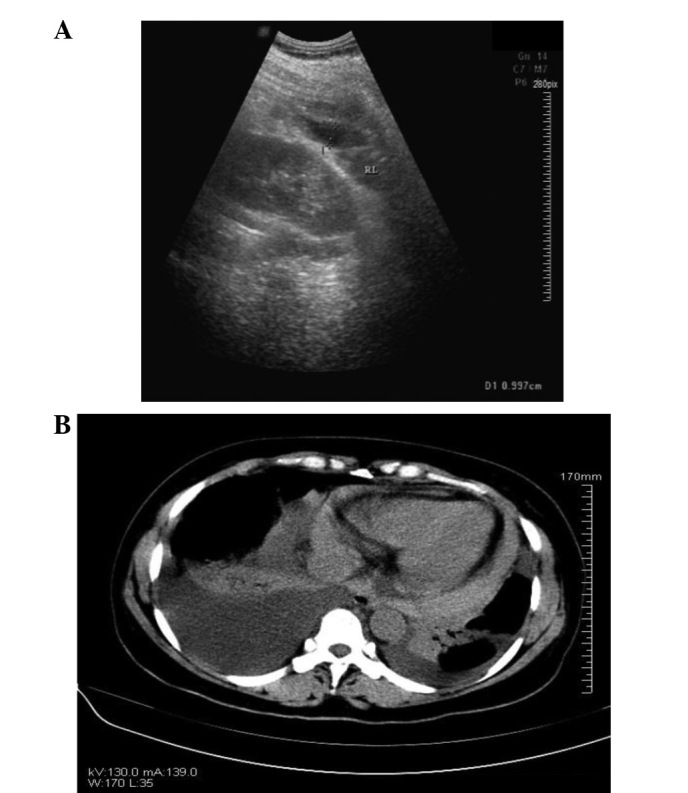
(A) Bedside B-mode ultrasound showing moderate amounts of bilateral pleural effusion and a small amount ofpericardial effusion. (B) CT indicating bilateral pleural and pericardial effusions.

**Figure 5:**
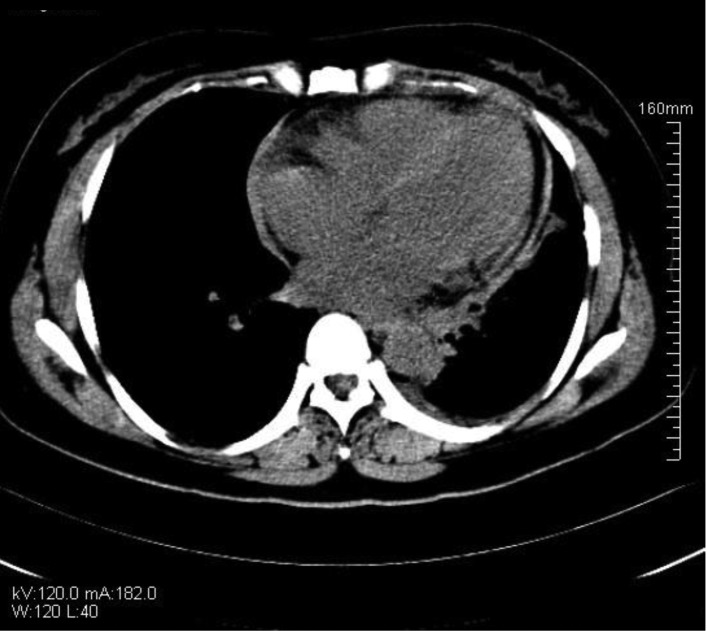
CT revealing a small amount of pericardial effusion and disappearance of the pleural effusion.

On post-operative day 4, the patient reported slight shortness of breath. On day 5, chest radiography (Fig. 3A) showed a small amount of bilateral pleural effusion and cardiac enlargement, however, the pre-operative chest radiograph (Fig. 3B) had not shown any cardiopulmonary abnormalities. On post-operative day 6, the patient underwent bedside color Doppler echocardiography (Fig. 4A) and chest CT (Fig. 4B). The results of these analyses indicated bilateral pleural and pericardial effusions. Diuretics, potassium and albumin supplements, hepatoprotective treatment and other treatments were applied. Following a consultation with the Department of Cardiothoracic Surgery, the patient received right pleural puncture and drainage after ultrasound localization and local anesthesia in the ward. The puncture was performed without complications and ~60 ml pale, yellow pleural effusion was drained. There was no bloody fluid. Following surgery, the patient reported that the shortness of breath had been significantly alleviated. The anti-inflammatory, hepatoprotective and symptomatic treatments were continued. On post-operative day 10, re-examination using chest and abdominal CT showed that the effusion had been significantly reduced (Fig. 5) and that the symptoms had improved substantially. The treatment was therefore considered effective.

The patient recovered and was discharged on post-operative day 16. During the year that followed, follow-up examinations showed that the patient’s condition had stabilized. Liver CEUS showed no recurrent space-occupying lesions and no recurrent symptoms, including pericardial effusion.

## Discussion

HCC is a common malignant tumor (6). Treatment of liver tumors has developed from radical surgery to comprehensive multidisciplinary treatment, involving surgery, intervention and chemotherapy (7). For HCC of small foci (diameter, <4 cm), the RFA method has the same result as surgical resection (8). Rossi *et al* were the first to successfully use RFA to treat liver tumors clinically (9). Following this, RFA gradually became one of the primary methods used for the local treatment of liver tumors. RFA is also used to treat tumors close to large blood vessels in the liver or complex foci in extrahepatic tissues (10).

In the current study, the patient exhibited post-operative pericardial effusion following RFA treatment. During the pre-operative ultrasound examination, an uneven weakened echo was located in a 16×16-mm area close to the liver capsule in the left upper hepatic segment. The results of CEUS were consistent with the diagnosis of small HCC. The lump was located at the liver capsule in the left second liver segment, close to the left diaphragm. Under local anesthesia, the patient received RFA treatment at the lump in the outer lobe of the left liver. Post-operative pericardial and pleural effusions were observed and the patient’s condition improved following symptomatic treatment. Post-operative complications after RFA treatment of liver tumors are not uncommon. However, they mainly include fever, pain, abdominal bleeding, bile duct injury, bowel injury, liver abscesses and implantation metastasis. There have been no previous reports of pericardial effusion (5).

To the best of our knowledge, this is the first report of post-operative pericardial effusion following RFA treatment. As the tumor was located at the liver capsule on the left hepatophrenic side close to the diaphragm, thermal conduction during RFA may have caused damage to the diaphragm and pericardium and this may have led to localized pericardial edema, which compressed the suprahepatic vena cava, eventually causing pericardial effusion. Following surgery, treatment measures, including diuretics, potassium and albumin supplements, hepatoprotective treatment and thoracentesis were applied promptly and the patient was re-examined using B-ultrasound and CT imaging in a timely manner. The patient eventually recovered well. A small amount of pericardial effusion remained, but there was no effusion in the thoracic or abdominal cavities and the AFP was decreased significantly. The patient was discharged from the hospital as their condition was stable.

This case emphasizes the possibility that adjacent tissues and organs may be damaged during RFA. To reduce the incidence of intraoperative and post-operative complications, the following recommendations must be noted: i) The position and extent of the tumor must be assessed carefully pre-operatively using imaging techniques. Using the position of the tumor relative to the portal vein and major intrahepatic bile ducts and its distance from major structures, such as the liver capsule, diaphragm, gallbladder and porta hepatis, the feasibility of RFA must be analyzed in strict accordance with the indications and contraindications for RFA. ii) For patients whose tumor location is complicated, particularly those whose tumors are near the diaphragm, such as in the present case, RFA must be performed under laparoscopy or by pneumoperitoneum to avoid diaphragmatic injury and heat transfer. iii) The optimal path for RFA must be selected based on the location of the tumor. For tumors located on the diaphragmatic side of the liver capsule, after RFA puncture needles are inserted into the tumor, they may be pulled slightly away from the diaphragm in order to avoid damaging it. iv) Depending on the patient condition, pre-operative TACE/TAE may be performed to reduce the tumor volume prior to RFA treatment. v) Adequate intraoperative analgesia must be applied to ensure patient compliance. vi) Adequate ablation boundaries must be ensured. vii) The proper RFA electrodes and frequency must be selected to avoid the overheating that causes damage to adjacent tissues and organs. viii) Prior to surgery, 200–500 ml saline (artificial ascites) may be injected into the space between the diaphragm and tumor focus to control the local temperature and prevent burning of the surrounding adjacent tissues and organs. ix) During and following the surgery, the patient’s vital signs must be monitored carefully. The post-operative re-examination should cover the chest, abdomen, pelvis and other areas and must not be confined to the abdominal cavity.

In summary, minimally invasive RFA treatment has become a preferred approach in the field of multidisciplinary comprehensive treatments of liver tumors and has come to play an increasingly significant role. In the future, during RFA treatment of liver cancer, precautions must be taken to precisely determine the position and extent of the RFA area to reduce damage and avoid post-operative complications, including those reported in this case.
